# Sulfasalazine Sensitizes Polyhematoporphyrin-Mediated Photodynamic Therapy in Cholangiocarcinoma by Targeting xCT

**DOI:** 10.3389/fphar.2021.723488

**Published:** 2021-08-13

**Authors:** Yan-Wen Zheng, Xiong-Ying Miao, Li Xiong, Bo Chen, Fan-Hua Kong, Jiang-Jiao Zhou, Zhong-Tao Liu, Yu Wen, Zi-Jian Zhang, Heng Zou

**Affiliations:** Department of General Surgery, The Second Xiangya Hospital, Central South University, Changsha, China

**Keywords:** cholangiocarcinoma, photodynamic therapy, sulfasalazine, solute carrier family 7 member 11, polyhematoporphyrin

## Abstract

Cholangiocarcinoma (CCA), which is highly malignant, shows a relatively poor prognosis, due to the insensitivity of the tumour to chemotherapy and radiotherapy. Photodynamic therapy (PDT) has become a promising palliative therapeutic option for patients with unresectable cholangiocarcinoma (CCA), while the functional amount of ROS is limited by intracellular redox systemen. Sulfasalazine (SASP), a well-known anti-inflammatory agent, which also acts as an inhibitor of the amino acid transport system xc (xCT), decreases the intracellular glutathione (GSH) level, thus weakening the antioxidant defence of the cell by inhibition of the antiporter. However, the combination of SASP and PDT remains unexplored. We have reported that polyhematoporphyrin (PHP)-mediated PDT inhibits the cell viability of CCA cells and organoids. Furthermore, in PHP-enriched HCCC-9810 and TFK-1CCA cells, SASP enhances the sensitivity to PHP-mediated PDT through a GSH-dependent mechanism. We found that PHP-PDT can up-regulate xCT expression to promote cells against overloaded ROS, while SASP reduces GSH levels. After the combination of SASP and PHP-PDT, cell viability and GSH levels were significantly inhibited. xCT was also observed to be inhibited by SASP in human organoid samples. Our findings suggest that, in combination with PDT, SASP has potential as a promising approach against CCA.

## Introduction

Cholangiocarcinoma (CCA) is an adenocarcinoma or papillary tumour arising from the bile duct epithelial cells, located along the intrahepatic and extrahepatic biliary tree (GERA et al., 2017; KENDALL et al., 2019). Epidemiology has shown that the morbidity and mortality of CCA have an uprising trend worldwide (BRIDGEWATER et al., 2016). CCA is more prevalent in Asia, with an incidence of up to 85 per 100,000 people in some areas (KHAN et al., 2019). Among many treatments, CCA is insensitive to chemotherapy and radiotherapy, with surgical resection still being the first treatment option (DOHERTY et al., 2017). However, the early stage of CCA has no evident clinical symptoms, and thus, patients usually lose the opportunity to undergo surgical operation after diagnosis (KRASINSKAS, 2018), contributing to a survival period of non-surgery patients lower than 1 year (RERKNIMITR et al., 2013). However, research shows that patients with CCA with effective palliative treatment can have a longer survival period (GAIRING et al., 2021). Endoscopic stent is a well-known palliative treatment for persistent drainage of the bile duct (CILLO et al., 2019). We urgently need to develop new methods to effectively control CCA.

With the continuous emergence and research on new medical technologies, photodynamic therapy (PDT) has shown brilliant potential for the treatment of CCA (BENSON et al., 2014). PDT is a treatment that involves the combination of three elements: photosensitizer (PS), molecular oxygen and light of a specific wavelength. The basic principle of the treatment is to generate a photochemical reaction under the excitation of the PS enriched in the tumour tissue by means of a certain-wavelength light to generate reactive oxygen species (ROS), such as excited singlet oxygen and superoxide negative radicals (ZHANG et al., 2020). PDT, together with biliary stenting, may improve the patency of the bile duct, by reducing the local obstruction caused by cancerous tissues, owing to the cytotoxic effects of ROS (LI et al., 2020a; HE et al., 2021). The most effective way to combat ROS in cells is to reduce such peroxides using glutathione (GSH). Many studies have reported that the level of GSH in tumours is much higher than that in normal tissues (YANG et al., 2019; LIN et al., 2020). This may be one of the reasons for their strong adaptability and an unfavourable factor for PDT in the treatment of CCA. Therefore, combined anti-GSH drugs may be beneficial to the success of PDT. Sulfasalazine (SASP) is widely used in the treatment of inflammatory bowel diseases and rheumatoid arthritis (DROSOS et al., 2020; SZYMAŃSKA et al., 2021). SASP also has an anti-tumour effect that can induce cytotoxicity by suppressing the specifical cystine/glutamate antiporter (amino acid transport system xc, xCT) in cancer cells (HARRIS and DENICOLA, 2020). Cysteine is a primary limiting precursor of glutathione (GSH) biosynthesis, and GSH is depleted during the accumulation of intracellular ROS to protect cells from oxidative damage (LV et al., 2019). Therefore, it is an excellent therapeutic strategy to enhance the sensitivity of tumours to PDT by clearing GSH. Jiang F *et al.* (JIANG et al., 2003) investigated buthionine sulfoximine, an agent which lowers the cellular glutathione level and enhances the photofrin-PDT treatment of human glioma.

Combining multifarious anticancer therapies has been the focus of recent research on the treatment of cancer. These combination modalities have various advantages, such as an enhanced curative efficacy, delayed drug-resistance, and reduced side effects. We propose that SASP-induced cystine depletion resulted in a reduction in the intracellular GSH levels, thus inducing a rapid accumulation of ROS, which become a steady resource for PDT. Therefore, we reason that a combined therapy may lead to remarkable cytotoxic effects compared to SASP or polyhematoporphyrin (PHP) PDT treatment alone in CCA. These observations will exploit the approach of repurposing SASP as a PDT sensitizer and anti-tumour therapeutic for the treatment of CCA.

## Materials and Methods

### Cell Lines and Reagents

RPMI-1640 was bought from Hyclone (Pittsburgh, CA, United States). Foetal bovine serum was purchased from BI (Beit HaEmek, Israel). A cell viability assay kit was purchased from Promega (Madison, Wisconsin, United States). An apoptosis detection kit (PI/Annexin V-FITC) and ROS assay kit (DCFH-DA) were purchased from Genview (Pompano Beach, FL, United States). The reduced glutathione assay kit was purchased from Jiancheng Bioengineering Institute (Nanjing, China). β-ME was purchased from MP Biomedicals (Santa Ana, CA, United States). SASP, NAC, and bovine serum albumin were purchased from Sigma (St Louis, MO, United States). Culture plates/flasks/dishes were acquired from Corning (Tewksbyry, MA, United States). Western blot analysis-related devices and reagents were acquired from Bio-Rad (Hercules, CA, United States). For immunohistochemistry staining, we used the rabbit two-step method kit (Nakasugi Jinqiao, PV-6001). The xCT and GAPDH primary antibody were purchased from Proteintech (Wuhan, Hubei, P.R.C, 26864-1-AP, 10494-1-AP). For the colony formation experiment, we used crystal violet (C0775, Sigma-Aldrich). The medium and lysate that were necessary for organoid culture were acquired from ACCURATE biotechnology.

### RNA Expression Information Acquisition and Analysis

We downloaded RNA expression data, the E-MTAB-6389 dataset with patient prognostic information from the European Bioinformatics Institute (EMBL-EBI) database (https://www.ebi.ac.uk/) (COOK et al., 2019). This dataset contained the following information: expression profiles obtained using array or high-throughput sequencing, age, histological type, and OS. The microarray platform used for the E-MTAB-6389 dataset was GPL17585. According to the annotation file provided by the platform, we used the “limma” package of R software to exclude the coding genes with missing values, annotated the RNA expression data one by one according to the gene name, and then used the log2 function to pre-process the gene expression values. For a gene containing multiple probes, the median was used to represent the expression value of the gene. The RNA expression data of the patients was visualized using heat maps and volcano maps, and xCT (*SLC7A11*) was labelled in the volcano map.

### Western Blot

RIPA was used to lyse the HCCC-9810 and TFK1 cells after DVDMS-PDT treatment to prepare the protein samples. The lysates were centrifuged at 16,000 × *g* for 5 min at 4°C in a suitable centrifuge tube. The supernatant containing the cell extract was transferred to a new tube, 5× SDS loading buffer was added and denaturation was performed at 95°C for 5 min. Before electrophoresis, the BCA method was used to analyse the protein concentration. The electrophoresis conditions used were: 120 V, 50 min. After electrophoresis, a PVDF membrane was used for protein transfer at 400 mA for 45 min. The PVDF membrane was blocked using non-fat milk prepared using TBST. Polyclonal anti-xCT or anti-GAPDH antibodies (all diluted at 1:1,000) were used for primary antibody incubation overnight. Then, the membrane was incubated with HRP-conjugated secondary antibody. Finally, the PVDF membrane was washed and developed using an ECL luminescent solution and photographed for archiving. ImageJ was used for the quantification or densitometry.

### Immunohistochemical Staining

We collected 34 pairs of tumour and para-tumour tissues from patients with cholangiocarcinoma in our hospital. All patients signed an informed consent, and the experiment was approved by the hospital ethics committee. We first embedded the tissue in paraffin and then sliced the tissues. The slides were sequentially incubated with xylene, alcohol-100%, alcohol-95%, alcohol-90%, alcohol-80% and alcohol-70% for dewaxing. Then, antigen retrieval was performed, the endogenous catalase was removed, and the antigen site was exposed. Serum was added to block some non-specific sites. The serum around the back and front tissues of the slides was dried using absorbent paper. After adding the primary antibody, the slides were stored overnight in a refrigerator at 4°C. The secondary antibody was added and the slides were incubated in a 37°C incubator for 30 min. The slides were taken out of the incubator, washed with PBS for three times and the developer was added. The developed tissue was soaked in hematoxylin and dyed for 30 s. The slide glass was sequentially incubated with alcohol-70%, alcohol-80%, alcohol-90%, alcohol-95%, alcohol-100%, alcohol-100% and xylene for dehydration. Neutral gum was added to the side of the tissue and covered with a cover slip at last. The immunohistochemical staining results were assigned a mean score considering both the intensity of staining and the proportion of cells with an unequivocal positive reaction. Positive reactions were defined as those showing brown signals mainly in the cell plasma or membrane. A staining index was determined by the staining intensity (values, 0–3) and positive area (values, 0–4). The scores were defined as staining intensity × positive area. For statistical analysis, scores of 0 were considered negative expression and scores of 1–12 considered positive expression.

### Cell Lines

CCA and bile duct epithelial cell lines HCCC-9810, RBE, TFK-1 and HIBEpiC were purchased from the Cell Resource Centre of Shanghai Institutes for Biological Sciences and maintained in RPMI-1640 medium supplemented with 10% foetal bovine serum. Cell lines were routinely cultured at 37°C, with 21% O_2_ and 5% CO_2_ and they were tested negative for mycoplasma before any drug treatments were conducted.

### Confocal Imaging Analysis

To measure the cell internalisation of PHP, HCCC-9810 cells were plated onto 35 mm confocal laser culture dishes for 24 h. After washing twice with PBS, the cells were incubated for different time periods with 20 μg/ml PHP in 1 ml of medium containing 10% FBS. After the medium was removed, the cells were washed twice with PBS, followed by staining with Gold Antifade Mountant with DAPI (SlowFade™, Thermo Scientific, S36942, United States), and visualized under a confocal laser scanning microscope. PHP was detected at an emission wavelength above 660 nm and an excitation wavelength of 405 nm, and Gold Antifade Mountant with DAPI was detected in the 430–500 nm emission range at an excitation wavelength of 405 nm. The cellular fluorescence images were collected using an FV 500-IX70 confocal microscope (Olympus America Inc. Melville, NY) with a ×100 objective.

### Photo Dynamic Therapy and Cell Viability

HCCC-9810 and TFK-1 cells were plated onto 6-well plates for 24 h. After washing twice with PBS, the cells were incubated with 0–40 μg/ml PHP in 2 ml of medium containing 10% FBS. After incubating in PHP for 4 h, we used a 630 nm wavelength laser to irradiate the 6-well plate until the total energy density reached 10 J/cm^2^.

HCCC-9810 and TFK-1 cells were seeded on 96-well plates at a density of 1.5 × 10^4^ cells/ml and cultured 24 h prior to drug treatment. Cell viability was determined using the Cell Alamar-Blue^®^ reagent (Promega, G8082, United States). A 37°C water bath was used to thaw the Cell Alamar -Blue^®^ reagent and bring it to ambient temperature. Assay plates were removed from the 37°C incubator, and 20 µl/well of Cell Alamar-Blue^®^ Reagent were added. The plates were shaken for 10 s. Cells were incubated in standard cell culture conditions for 2 h. The plates were shaken for 10 s, and the fluorescence intensity was recorded at 560/590 nm.

### Microarray Analysis After Photo Dynamic Therapy Treatment

First, PDT was used to treat the RBE cell line (10 J/cm^2^). The total RNA of the sample was extracted using TRIZOL. The total RNA of the sample was quantified using NanoDrop 2000 (Thermo Scientific) and the RNA integrity was checked using Agilent Bioanalyzer 2100 (Agilent Technologies). The Agilent Human lncRNA Microrray V6 (4 × 180 K, Design ID: 084410) chip was used to detect the expression levels of the cell samples. After the samples were subjected to quality control, the data were standardized using quantile regression. The standardized data was filtered, and in each group of samples used for comparison, at least one group in which 75% of the samples were marked as detected probes were left for subsequent analysis. The differential genes were screened using *p*-value and fold-change values in the *t* test. At the same time, heatmap and volcano plots were drawn using the results of the differential expression genes. The original image extraction and original data acquisition of the chip were completed by OEBIOTECH company, and the drawing and enrichment analysis were performed using the R script.

### Colony Formation Experiment

HCCC-9810 and TFK-1 cells were seeded on a 6-well plate at a density of 2,000 cells/well. After 72 h, the cholangiocarcinoma cells were subjected to PDT and/or SASP treatment. On the 10th day, the medium was removed from the wells, the cells were washed twice with PBS, fixed in a 4% paraformaldehyde (PFA) incubator for 10 min, stained with a crystal violet solution for 10 min, and washed twice with PBS. Photos of the colonies in the wells were taken using a digital camera (iphone X).

### Annexin-V-FITC/PI Assay

Briefly, cells that had underwent different drug treatments were harvested and resuspended in binding assay buffer, and then stained with Annexin-V-FITC and PI for 15 min at room temperature in the dark. The apoptosis of cells was determined by using flow cytometer (Beckman Coulter Epics Altra, Miami, FL) analysis.

### Analysis of the Cellular Glutathione and Reactive Oxygen Species Levels

The intracellular GSH levels were analysed using the reduced glutathione assay kit (Nanjing Jiancheng Bioengineering Institute, Nanjing, China) according to the manufacturer’s instructions.

To measure the intracellular ROS levels, cells were incubated with 10 μM DCFH-DA (Sigma-Aldrich, St. Louis, MO, United States) in the dark for 30 min. The stained cells were collected and resuspended in 1× PBS, and then subjected to flow cytometer (Beckman Coulter Epics Altra, Miami, FL, United States) analysis.

### Organoid Culture

Samples for organoid culture were collected from five patients with CCA. Specimens of at least 1 cm^3^ were collected and 4–5 ml of a preheated human tissue digestion solution were added per gram of specimen, and the mixture was digested in a shaker at 37°C for 2 h, followed by washing and passing through a 70 μm nylon filter and the collected cell suspension was centrifuged at 8°C and 300 × g for 5 min. The supernatant was discarded and the procedure was repeated 3–5 times. After counting the cells, the 1000 cells were resuspended in 50 μl of Matrigel, and a drop of BME2 was added to the centre of 12 well plates to seed the cells. After plating, the medium was changed every 2–3 days, on average. Primary organoids need to be passaged after 5–7 days of culture. The passage method is basically the same as that of the cell line but it does not require the use of pancreatin. The detailed method is described in our previous literature (HUANG et al., 2021).

### Photo Dynamic Therapy, Viability Detection and Sectioning of Organoids

After the organoids were successfully extracted and cultured for 3–5 days, a special organoid medium containing 20 μg/ml PHP and/or 500 μM SASP was prepared. After 72 h, the 6-well plate was irradiated with a laser at 630 nm until the total energy density reached 10 J/cm^2^. PI was used at a final concentration of 5 μg/ml and Hoechst 33,342 dye was used at a final concentration of 5 μg/ml to dye the organoids. The survival status of cells in the organoids was recorded.

IHC was performed on organoid samples treated with PDT and SASP. A single suspended cholangiocarcinoma organoid was obtained by removing the Matrigel, fixing with 4% PFA, washing with PBS-B, and resuspending in 70% ethanol. After dehydration using 100% ethanol, the organoids were marked with eosin staining. After being washed with xylene, the organoid cell clusters were embedded in paraffin and dried. After organoid sectioning, conventional IHC was performed for xCT detection.

### Statistical Analysis

In this study, SPSS^©^ Statistics 25 and GraphPad Prism 8 were used for statistical analysis and drawing, and RNA microarray data was analysed using R 3.6.2. All quantitative data were presented as mean ± SEM and obtained from, at least, three independent experiments. Analysis of variance was performed on the data using ANOVA test. Levin’s variance equality test and two-sided *t* test were used for the two independent samples. Survival analysis uses logrank test. CompuSyn programme was used to analysis the combination effect. *p* < 0.05 is statistically significant, and *p* < 0.05 is annotated with *, and *p* < 0.01 is annotated with **.

## Results

### xCT Increases the RNA and Protein Levels in Cholangiocarcinoma Patients and Cell Lines

**FIGURE 1:**
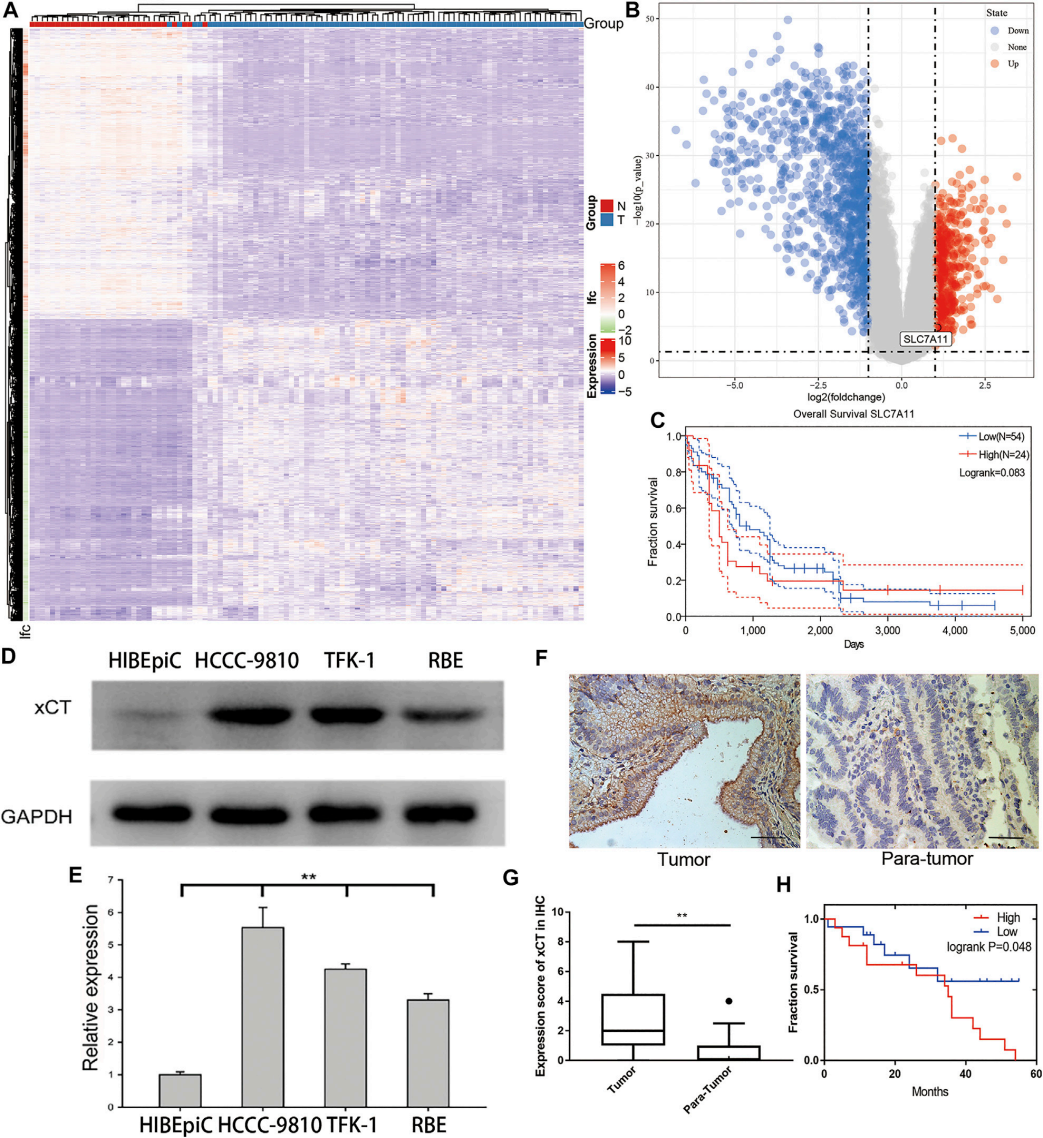
The expression of xCT in cholangiocarcinoma and its relationship with the prognosis of patients. **(A,B)** Differential gene heat map and volcano map from the E-MTAB-6389 dataset which include 78 tumour (T group) and 31 adjacent tissue (N group) samples with high and low expression; xCT (*SLC7A11*) is marked. **(C)** Survival analysis of xCT in the E-MTAB-6389 dataset. **(D,E)** xCT expression levels and histograms in normal bile duct epithelium HIBEpiC cells and cholangiocarcinoma HCCC-9810, TFK-1 and RBE cells. **(F,G)** The represent images and histogram of xCT in cholangiocarcinoma tumour and adjacent tissues (34 pairs). **(H)** Survival analysis showed the effect of different expression levels of xCT on the survival time of patients with cholangiocarcinoma (High, high expression group; Low, low expression group).

To determine whether there are differences in the expression of xCT between the tumour tissues and adjacent tissues of patients with CCA, we first analysed the RNA expression matrix of the E-MTAB-6389 dataset. The dataset includes 78 tumour (T group) and 31 adjacent tissue (N group) samples. After clustering the different genes in the sample, the tissues and genes can be roughly divided into high and low expression groups (Figure 1A). Through screening of differential expression multiples and *p*-values, it was found that *SLC7A11* (xCT) was significantly up-regulated [log(fold-change) = 1.071, adjP <0.001] (Figure 1B). Taking the average of the xCT expression values as the critical value, survival analysis of 78 patients with CCA showed that the overall survival (OS) of patients with a high xCT expression was worse than those with a low xCT expression, *p* = 0.083 (Figure 1C).

To verify the results of this dataset, we used western blot experiments to compare the xCT expression of normal bile duct epithelial HIBEpiC cells and CCA cell lines HCCC-9810, TFK-1 and RBE. The results showed that xCT expression was higher in CCA cell lines (Figures 1D,E). The immunohistochemical staining (IHC) results of 34 matched tumours and adjacent tissues also indicated that xCT protein was expressed more strongly in tumours (Figures 1F,G). More importantly, in the patient we collected, high expression of xCT was significantly associated with worse prognosis. According to the expression level of xCT, HCCC-9810 and TFK-1 cells were used for subsequent experiments.

### Effect of Polyhematoporphyrin-Photo Dynamic Therapy on the Cytotoxicity of HCCC-9810 and TFK-1 Cells

**FIGURE 2:**
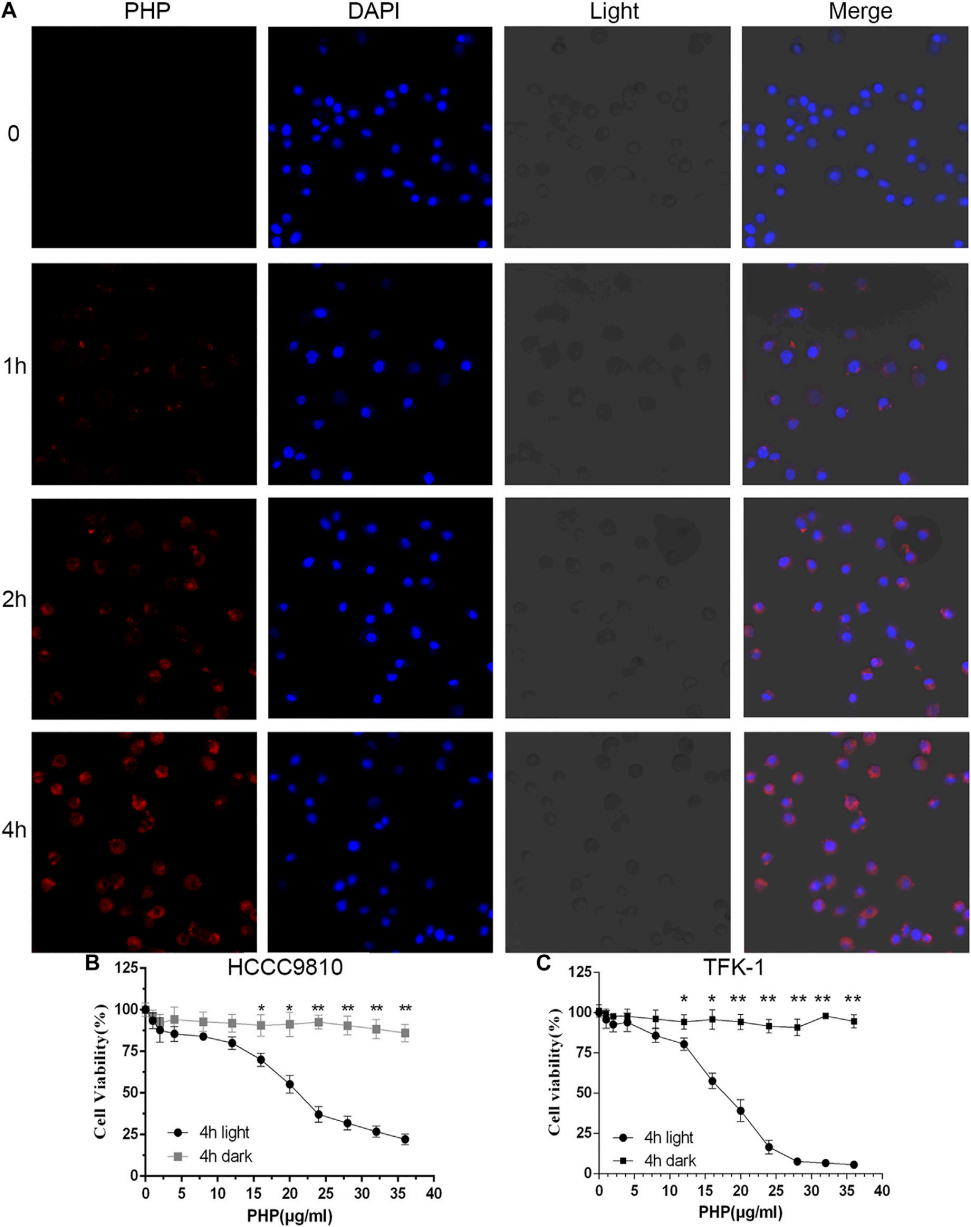
Phototoxicity of CCA cells. **(A)** Representative CLSM images demonstrating the cellular uptake of PHP (red) with HCCC-9810 cells after incubation with PHP for 0–4 h at 37°C. Original magnification: 400×. **(B,C)** Relative cell viability of PHP-treated HCCC-9810 and TFK-1 cells in the dark or with light irradiation at 630 nm (10 J/cm^2^). Data are shown as mean ± SEM (*n* = 3). *p < 0.05, **p < 0.01.

The premise of PDT-induced cytotoxicity is the effective endocytosis of the PS. We determined whether HCC-9810 and TFK-1 cells can uptake PHP. We found that a time-dependent increase in the drug uptake was detected using confocal laser scanning microscopy (CLSM). PHP, when added at a final concentration of 20 μg/ml at 37°C for 4 h, could efficiently get into the HCCC-9810 and TFK-1 cells (Figure 2A). We further detected the inhibitory effect of PHP-mediated PDT (PHP-PDT) on HCCC-9810 and TFK-1 cells. Cells were treated for 4 h with different concentrations of PHP and subjected to laser irradiation (10 J/cm^2^), and then cell viability was measured *via* Alamar-Blue assay. We found that PHP-mediated PDT could reduce cell viability in a dose-dependent manner (Figures 2B,C).

### Polyhematoporphyrin-Photo Dynamic Therapy Promotes the Rise in xCT RNA and Protein Expression

**FIGURE 3:**
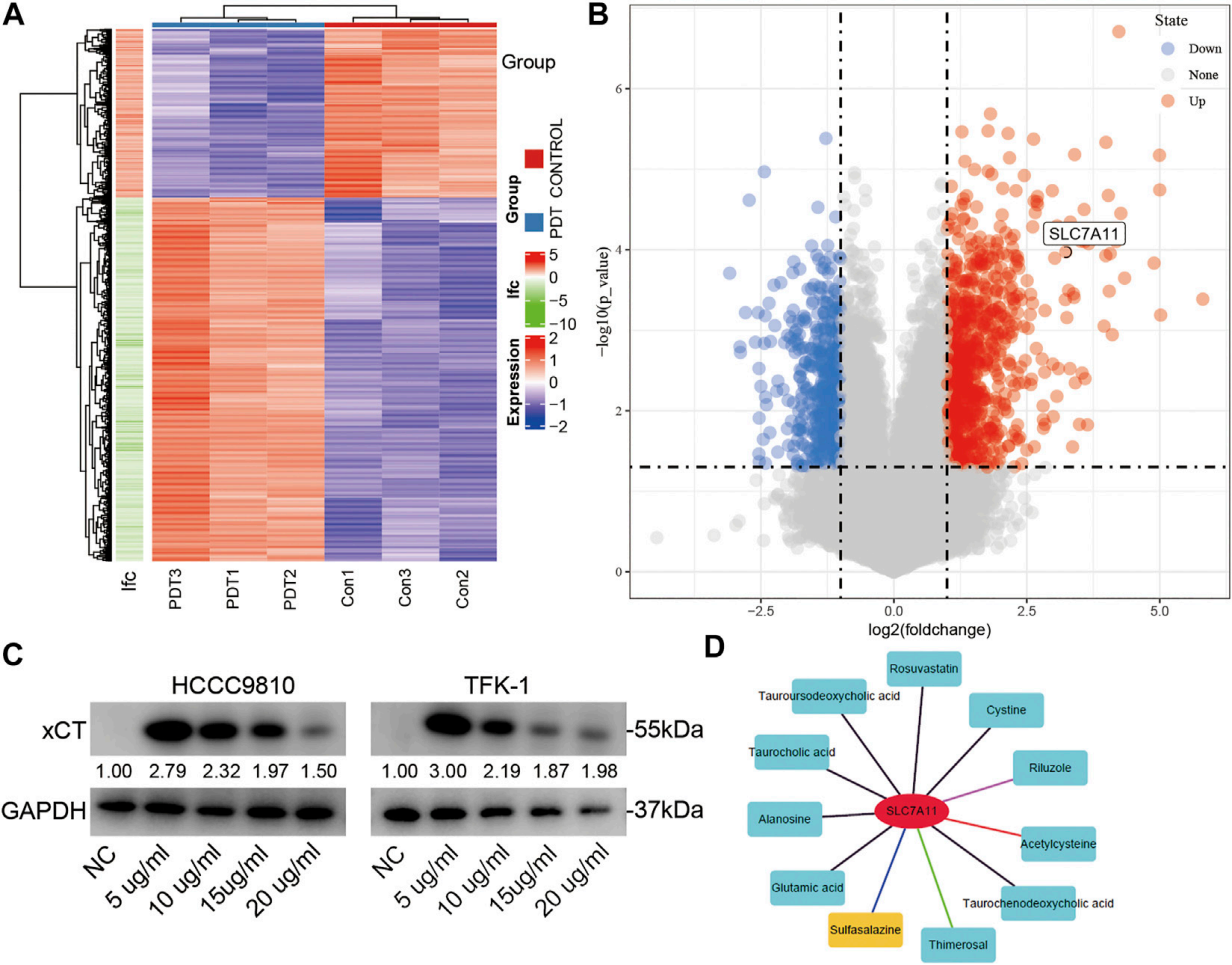
The expression of xCT changes after PDT treatment. **(A,B)** The heatmap and volcano map of the differential genes after PDT treatment; xCT (*SLC7A11*) is marked. **(C)** After treatment with different concentrations of PHP-PDT, the expression of xCT in HCCC-9810 and TFK-1 cells was different. **(D)** Potential drug targets of xCT.

PHP-PDT has a relatively obvious killing effect on CCA cell lines, but what role of the xCT plays in it is not yet known. Therefore, we first detected the changes in RNA expression after PDT treatment using an RNA microarray. After deduplication, a total of 1,086 genes were up-regulated or down-regulated after PDT treatment, and *SLC7A11* (xCT) was significantly up-regulated (log(fold-change = 3.234, adjP <0.001) (Figures 3A,B). After different concentrations of PHP-PDT treatment were used, the expression of xCT in HCCC-9810 and TFK-1 cells was also different (Figure 3C). Interestingly, with the increase in PHP concentration, the expression of xCT began to decrease, but its expression was always higher than that of the untreated group, which may indicate that GSH is gradually depleted, despite the increase in the ROS levels. The combined results of the aforementioned up-regulation of xCT and the shorter OS suggest that xCT may be a resistance factor to PDT. Therefore, we found that SASP can be used as a drug targeting xCT through the drug-gene interaction database (https://dgidb.org/) (Figure 3D). The inhibition of xCT expression has been reported in Miyamoto’s work (MIYAMOTO et al., 2020).

### Sulfasalazine Promotes Polyhematoporphyrin-Photo Dynamic Therapy to Inhibit Cholangiocarcinoma Cell Viability and Increase Cholangiocarcinoma Cell Apoptosis

**FIGURE 4:**
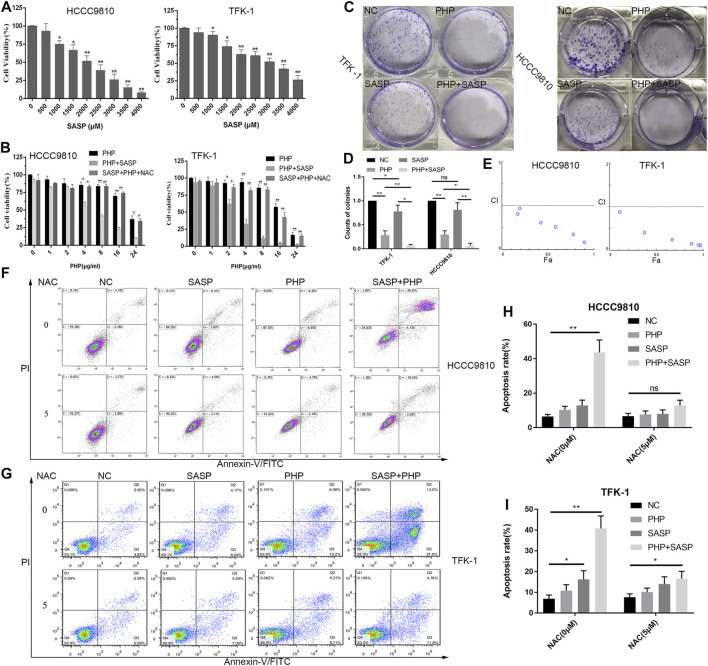
SASP confers enhanced sensitivity to PDT to CCA cells. **(A)** Cell viability of HCCC-9810 and TFK-1 cells was analysed by using Alamar Blue analysis after treatment with various concentrations of SASP. The mean value was calculated using a *t* test (mean ± SEM; *n* = 3). **(B)** Cell viability of HCCC-9810 cells analysed by using Alamar-Blue analysis after treatment with various concentrations of PHP-PDT and their combination with SASP (0.75 mM) in the presence or absence of N-acetyl-L-cysteine (NAC). **(C)** The colony formation of TFK-1 and HCCC-9810 cell lines after different interventions in the control group (NC), PHP-PDT group (PHP, 20 μg/ml, 10 J/cm^2^), SASP group (SASP, 750 μM) and PHP-PDT combined with SASP group (PHP + PDT). **(D)** The histogram shows the ability of colony formation in different groups. **(E)** Combination index (CI) of the use of various concentrations of PHP-PDT (1, 2, 4, 8, 16 and 24 μg/ml; 10 J/cm^2^) and SASP (0.75 mM) in HCCC-9810 and TFK-1 cells analysed using CompuSyn software. CI > 1 indicates an antagonistic effect; CI = 1 indicates an additive effect; CI < 1 indicates a synergistic effect. **(F,G)** After the same treatments used in **(C)**, the cells were detected using Annexin V-FITC/PI staining and flow cytometry. *p < 0.05, **p < 0.01.

Various studies have shown that SASP can induce GSH depletion, and thus lead to cell apoptosis (MONTELEONE et al., 2021). However, there are few studies on the use of SASP in CCA treatment. Therefore, the effect of different concentrations of SASP (0–4,000 μM) on the reduction of CCA cell viability was confirmed (Figure 4A) at first. The results showed that the anti-CCA effect of SASP is dose-dependent, and the maximum inhibitory concentration (IC50) value is 2.1 mM in both CCA cell lines. To further determine the efficacy of the combination of PHP-PDT and SASP, we used PHP-PDT (0–24 μg/ml) and SASP (750 μM), alone or in combination, to treat HCCC-9810 and TFK-1 cells to verify the possible synergistic toxic effects (Figure 4B). At this concentration, SASP alone cannot significantly inhibit the activity of CCA cells. The results showed that the PHP-PDT combined with SASP treatment group had a significantly lower cell viability compared with the drug treatment group alone. Moreover, N-acetyl-L-cysteine (NAC) can alleviate, but not reverse, the anti-CCA effect of this combination therapy group (Figure 4B). Colony formation experiments more intuitively confirmed the viability inhibition effect of PHP-PDT (20 μg/ml, 10 J/cm^2^) and SASP (750 μM) alone or in combination (Figures 4C,D). In addition, the median effect analysis programme (CompuSyn) was used to calculate the combined index of PHP-PDT and SASP to confirm the synergy of PHP-PDT and SASP. The combination index was lower than 1.0, indicating a synergy between PHP-PDT and SASP in HCCC-9810 and TFK-1 cells (Figure 4E). Through the Annexin V-FITC assay, it was confirmed that the combined use of PHP-PDT (12 μg/ml, 10 J/cm^2^) and SASP (750 μM) in HCCC-9810 and TFK-1 cells can yield stronger apoptosis induction results than either treatment alone (Figures 4F–I). These results indicate that SASP can make CCA cells sensitive to PDT.

### Sulfasalazine Significantly Increases the Levels of Reactive Oxygen Species in Cells Induced by Polyhematoporphyrin-Photo Dynamic Therapy by Down-Regulating the Glutathione Levels

**FIGURE 5:**
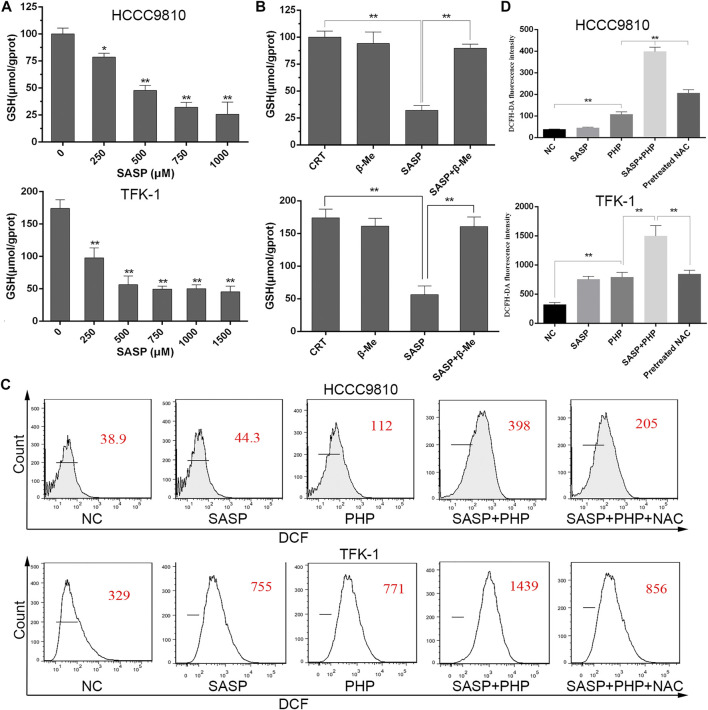
SASP down-regulates the GSH level in CCA cells. **(A)** Intracellular GSH level in HCCC-9810 (upper panel) and TFK-1 (lower panel) cells after treatment with various concentrations of SASP. **(B)** Intracellular GSH level in HCCC-9810 (upper panel) and TFK-1 (lower panel) cells after SASP treatment in the presence or absence of β-ME. **(C,D)** Flow cytometry chart and histograms of ROS levels after treatment with SASP (0.5 mM), PHP-PDT (12 μg/ml, 10 J/cm2) and their combination in HCCC-9810 and TFK-1 cells, respectively. Pre-treatment with 5 mM N-acetyl-L-cysteine (NAC) significantly reduced the ROS levels induced by the combination treatment. *p < 0.05, **p < 0.01.

To clarify whether SASP can deplete GSH in CCA cells, and thereby sensitize PDT, HCCC-9810 and TFK-1 cells were treated with different concentrations of SASP (0–1,500 μM) for 48 h. As the concentration of SASP increases, the intracellular GSH levels in HCCC-9810 and TFK-1 cells decrease (Figure 5A). β-ME has a protective effect against oxidative stress, and the effect of β-ME is related to the promotion of cystine uptake [17]. Therefore, the role of SASP in GSH consumption was evaluated by using β-ME. HCCC-9810 and TFK-1 cells were treated with SASP (750 μM), β-ME (66 μM) and SASP combined with β-ME for 48 h, respectively. Compared with the SASP treatment group, β-ME reversed the changes in GSH depletion induced by SASP (Figure 5B).

Based on this, the theoretical basis of the synergistic effect of the joint application of PHP-PDT and SASP is to generate more ROS. We used the ROS probe DCFH-DA to detect the levels of ROS when PHP-PDT (12 μg/ml, 10 J/cm^2^) and SASP (750 μM) were used alone or in combination (Figures 5C–F). The results showed that, compared with the drug treatment group alone, the ROS levels of the combination treatment group were significantly increased. In addition, in the presence of NAC, the effect of ROS production in the combined treatment group was impaired.

### The Effect of Sulfasalazine Combined With Polyhematoporphyrin-Photo Dynamic Therapy in Human-Derived Organoids Is Significantly Stronger Than the Either Treatment Alone

**FIGURE 6:**
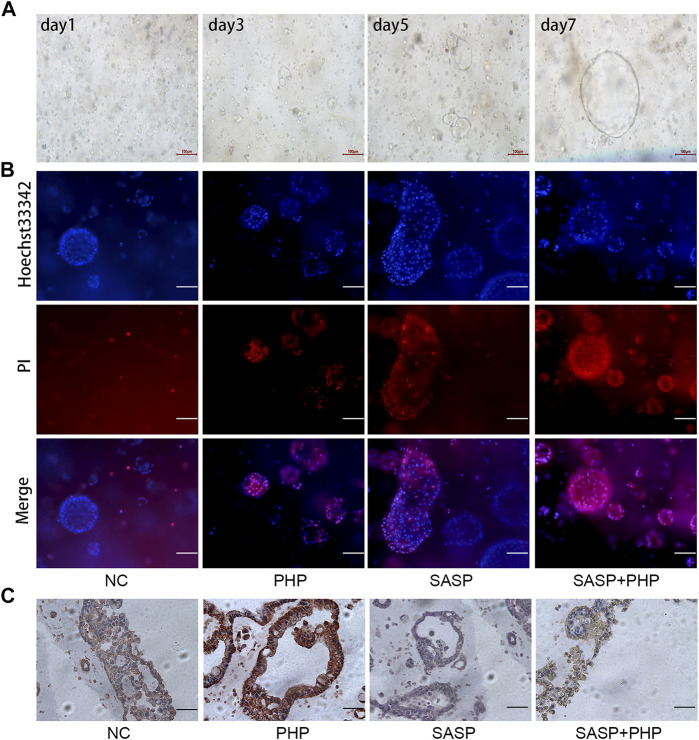
SASP sensitizes the anti-tumour effect of PHP-PDT in cholangiocarcinoma organoids. **(A)** The growth of organoids on days 1, 3, 5 and 7. **(B)** Hoechst 33,342 and PI were used to label the live and dead cells, respectively, of NC, PHP-PDT, SASP and the combination group. **(C)** The changes in xCT expression in cholangiocarcinoma organoids after SASP and PHP-PDT treatment alone or in combination.

The ultimate goal of using SASP for synergy with PHP-PDT is apply the treatment to clinical settings. Compared with the nude mouse subcutaneous tumour model, the organoids derived from patients better represent the sensitivity of human CCA to specific therapies. In this study, patient-derived CCA organoids were collected and cultured. It can be observed that the organoids grew rapidly from the granular cell clusters on the first day after extraction to a round follicular structure (Figure 6A), indicating that they are in a good condition and can be used for subsequent evaluation of combination therapy. After using PHP-PDT (20 μg/ml) and SASP (750 μM) to treat the organoids grown for 5 days, Hoechst 33,342 was used to distinguish the surviving or mildly apoptotic organoid cells, and propidium iodide (PI) was used to label the severely apoptotic or necrotic cells. The results show that PHP-PDT and SASP alone can only slightly induce apoptosis in CCA organoids, but the apoptosis effect is significant after the combination of the two (Figure 6B, Supplementary Figure S1).

### Sulfasalazine can Reverse the Increase in xCT Protein Expression After Polyhematoporphyrin-Photo Dynamic Is Used to Treat Human Organoids

Based on the verification of SASP sensitization using PHP-PDT in CCA organoids, we further intended to verify the changes in xCT expression. IHC was used to detect xCT expression in CCA organoids treated with SASP and PHP-PDT, and the results showed that the xCT expression of untreated CCA organoids (NC) was mild to moderate, and that of CCA organoids treated with PHP-PDT alone was strongly positive, while it was negative after SASP treatment alone. The xCT expression after the PHP-PDT + SASP treatment was weaker than that after PHP-PDT treatment alone, which was similar to the results obtained for the untreated CCA organoids (Figure 6C).

## Discussion

Cholangiocarcinoma is a malignant tumour that is difficult to be diagnosed early and is associated with a high mortality (BERTUCCIO et al., 2019). The effect of palliative treatments (radiotherapy, chemotherapy and stent) on unresectable CCA is still unsatisfactory (SUMIYOSHI et al., 2018). Therefore, finding feasible CCA treatment targets and methods is the only way to improve the prognosis of patients. xCT is a transporter subunit of cysteine and is often considered one of the marker proteins against ROS (CRAMER et al., 2017). In this study, we first analysed the RNA expression level of xCT in patients with CCA in the public dataset E-MTAB-6389 and its relationship with prognosis, and further detected the xCT protein expression in 34 pairs of samples from patients with CCA and in four types of bile duct cells. The results show that xCT is highly expressed at both the RNA and protein level in tumour tissues, and the public databases suggest that xCT can be used as a predictor of CCA prognosis. The expression of xCT in human tissues has also been reported for prognosis in bladder cancer (OGIHARA et al., 2019), hepatocellular carcinoma (KINOSHITA et al., 2013), and breast cancer (TIMMERMAN et al., 2013). Otsuki determined that Oxy, which is a vasodilator, is a sensitizer of xCT inhibitors, and that its GSH depletion-mediated sensitivity is beneficial to tumour ROS treatment (OTSUKI et al., 2020). Yu *et al.* (LI et al., 2020b) used cisplatin (CDDP)-resistant non-small cell lung cancer cells (N5CP cells) and found that CDDP-mediated activation of the xCT pathway is related to the resistance of cells to CDDP, while ergot alkaloid or sorafenib can increase the sensitivity of tumour cells to CDDP by inhibiting xCT pathway, and the two combined with low-dose CDDP can more effectively inhibit the growth of N5CP cells. Although there are currently few studies on xCT in patients with CCA, and patient-derived samples have not been subjected to multivariate analysis to confirm the independent prognostic effect of xCT, based on the existing evidence, we can also speculate that CCA showing a high expression of xCT has a high malignant potential.

PDT has been reported to have a favourable outcome as palliative therapy for unresectable CCA (MOOLE et al., 2017; GONZALEZ-CARMONA et al., 2019). Our results are consistent with previous reports (WU et al., 2017) revealing that PDT has a strong antitumor effect on CCA cells. The results demonstrate that PHP could accumulate rapidly in HCCC-9810 and TFK-1 cells and induce photochemical damage after laser irradiation, thus killing cancer cells. Unfortunately, we found that xCT is significantly up-regulated after PHP-PDT treatment in CCA cells, indicating that it may be a key protein for tumour resistance to ROS. The application of PHP-PDT in CCA relies on the generation of ROS. Therefore, generating more ROS in tumour tissues with limited oxygen is one of the key issues in improving the efficacy of PDT. Many PDT-related nanomaterials are developed on the theoretical basis of increasing the oxygen content in tissues and reducing the GSH levels (LIANG et al., 2018; XU et al., 2020). Therefore, this is a feasible method to reduce or consume autologous reducing substances. SASP is a very commonly used clinical drug that can directly inhibit xCT.

SASP is an effective inhibitor of xCT and was first reported to be effective in lymphoma cells. Recently, more and more studies have reported that SASP-mediated xCT down-regulation results in a variety of tumour suppressive effects in lung cancer (JI et al., 2018), gatric cancer (ZHUANG et al., 2021) and pancreatic cancer (HUNG et al., 2020). In addition, SASP can improve the efficacy of chemotherapy (SUGIYAMA et al., 2020). Recently, it has been discovered that CD44 interacts with xCT by binding to the surface of tumour cells. This interaction stabilizes the xCT on the cell membrane, thereby promoting the uptake of cysteine by the cell, which in turn promotes the synthesis of GSH (OGIHARA et al., 2019). GSH is a major antioxidant and is essential to protect cells from ROS. CD44 is one of the biomarkers of CCA (WATTANAWONGDON et al., 2019); thus, we believe that SASP targeting xCT will have a significant sensitization effect on PDT for the treatment of CCA. This study explored the effect of the combination of SASP and PHP-PDT on CCA cells. After the combined application of SASP and PDT, CCA cell apoptosis increased significantly. Furthermore, xCT-dependent ROS defence can make cancer cells tolerate various oxidative stress treatments. Chen *et al.* (CHEN et al., 2020) found that cells with a low expression of xCT are more sensitive to glucose deficiency, and SASP reduces the levels of ROS and cell death, which are increased by glucose deprivation by inhibiting xCT. SASP will produce anti-inflammatory and mild immunosuppressive effects when applied to the digestive tract. Therefore, clinical application of large doses of SASP may be not conducive to tumor therapy. Based on this consideration, we also only use small doses of SASP (750 μM) to combine with PDT. Fortunately, this study clearly shows that SASP can reduce the levels of intracellular glutathione, which promotes the further production of ROS and death of cells after PHP-PDT treatment.

Previous studies have reported that SASP makes cancer cells sensitive to other ROS-producing treatments. Ma *et al.* (MA et al., 2015) investigated the effect of combining SASP and cisplatin for treating colorectal cancer cells and found that SASP can enhance the intracellular platinum level and cytotoxicity of cisplatin. Lin *et al.* (MONIRUZZAMAN et al., 2018) investigated the effects of SASP on cold atmospheric helium plasma and on X-irradiation-induced apoptosis in human leukemic cells and elucidated the mechanism of apoptosis enhancement. CDDP can also be used to induce cells to produce ROS and CDDP-resistance can be reversed by SASP via targeting xCT and CD44v9, which may be the effective theoretical basis for the aforementioned research (WADA et al., 2018). CD44v is also one of the markers of cholangiocarcinoma. Some of the aforementioned documents have confirmed the interaction between xCT and CD44v (WADA et al., 2018). Therefore, we speculate that cholangiocarcinoma cells are more sensitive to ROS, and due to the presence of CD44v, more xCT can be inhibited by SASP. This may be the key reason why PDT up-regulates xCT so significantly in HCCC-9810 and TFK-1 and SASP can increase the efficacy of PDT. Our results clearly show that the combination of SASP and PDT significantly promoted the production of ROS in HCCC-9810 and TFK-1 cells. We have also established a human-derived organoid model of extrahepatic cholangiocarcinoma. The combined use of SASP and PHP-PDT also showed a significant inhibitory effect. This is the first time that the combined efficacy of SASP and PDT in a CCA organoid model has been shown. Compared with mouse transplantation models, organoids may be more able to demonstrate that the combination of SASP and PHP-PDT is a new method for the treatment of CCA. A case report has recently described a patient with metastatic CD44v9+ bladder cancer. After multidisciplinary treatment, including CDDP-based chemotherapy and SASP combined therapy, a complete curative effect was achieved (OGIHARA et al., 2019). Since SASP is a common drug used for digestive system disorders, and that PDT has been included in the NCCN clinical guidelines (BENSON et al., 2014), the safety of the two is guaranteed to a certain extent. Therefore, in the future, clinical application research of SASP combined with PHP-PDT is very likely to be carried out and confirmed.

The lack of detailed molecular mechanism research is one of the limitations of this article. Although xCT has recently been very a hot topic in anti-tumour research related to ferroptosis, the core contribution of this article is the exploration of whether a combination of drugs targeting xCT and PDT has a sensitizing effect. We are more concerned about whether this solution can be quickly applied to clinical settings. In addition, the samples we collected have not been subjected to xCT-related survival analysis, which is mainly limited by the short follow-up time and the small number of samples. We will hopefully assess these issues in future research.

In summary, for the first time, our present studies provide evidence that SASP inhibits cell viability through depleting GSH and conferring enhanced sensitivity to photodynamic therapy in CCA. Since the pharmacology and tolerability of SASP is well defined, it can enter clinical trials rapidly as an adjuvant. However, the cell lines often fail to completely recapitulate the true characteristics of CCA in clinical patients. Therefore, a clinical trial is necessary to find out whether combination therapy can improve the prognosis of patients with CCA.

## Data Availability

The datasets E-MTAB-6389 for this study can be found in the European Bioinformatics Institute (EMBL-EBI) database (https://www.ebi.ac.uk/arrayexpress/experiments/E-MTAB-6389/).

## Supplementary Material

Supplementary Figure 1 Histogram of PI and Hoechst33342 in organoids.

Supplementary Table 1 Different expression genes between CCA and paratumoral tissue.

Supplementary Table 2 Different expression genes between PDT treatment and control group.

Supplementary Data 1 Microarray rawdata for gene expression of PDTgroup and control group.
