# Supplementary figures and images for: Sulfasalazine Sensitizes Polyhematoporphyrin-Mediated Photodynamic Therapy in Cholangiocarcinoma by Targeting xCT
Source: Front Pharmacol. 2021 Aug 13;12:723488. doi: 10.3389/fphar.2021.723488 (PMC8414975; doi:10.3389/fphar.2021.723488)

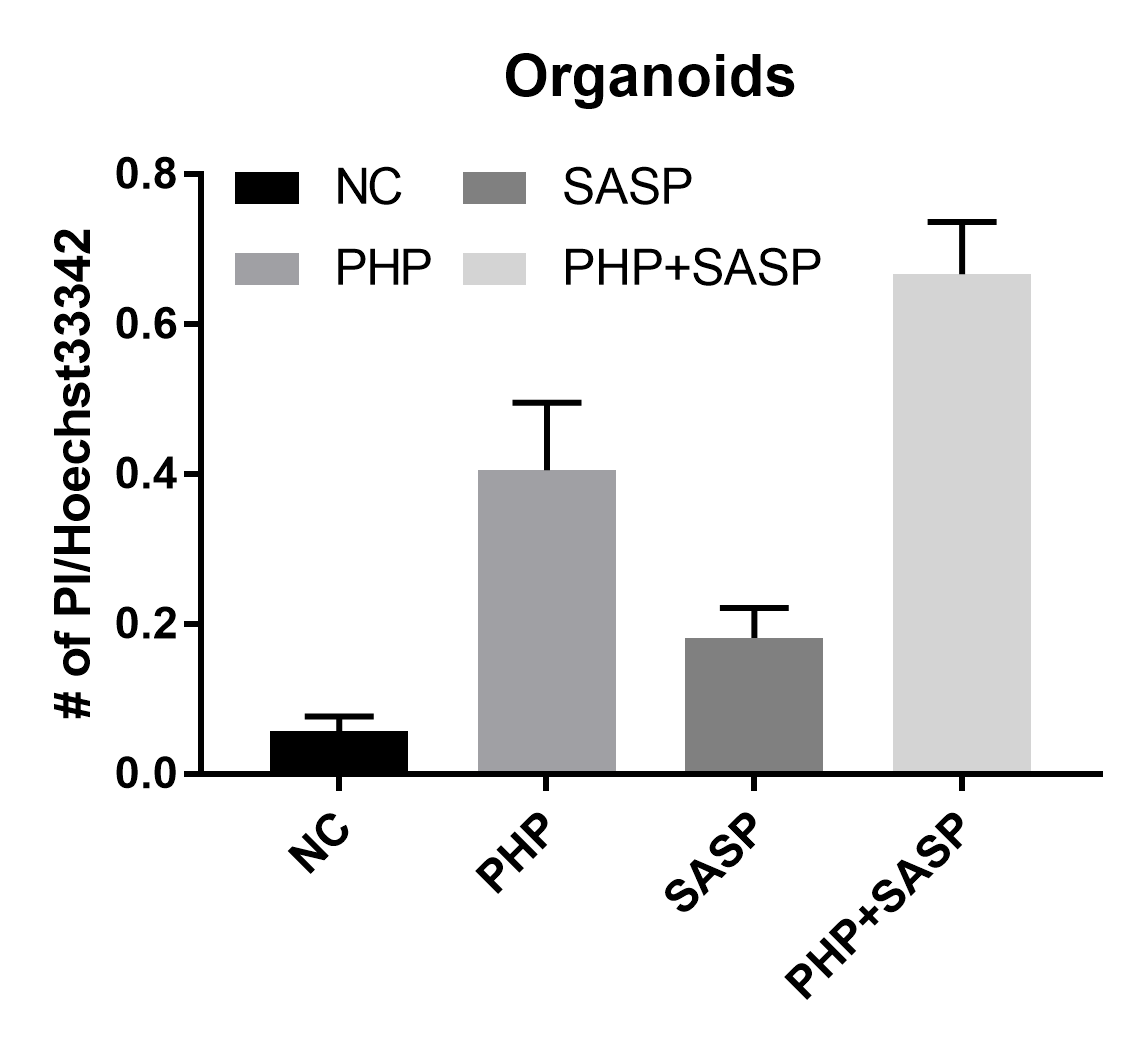

Supplement: Supplementary file 1 [file Image1.TIF]
